# Microbial Translocation Is Associated with Extensive Immune Activation in Dengue Virus Infected Patients with Severe Disease

**DOI:** 10.1371/journal.pntd.0002236

**Published:** 2013-05-23

**Authors:** Cornelia A. M. van de Weg, Cláudio S. Pannuti, Evaldo S. A. de Araújo, Henk-Jan van den Ham, Arno C. Andeweg, Lucy S. V. Boas, Alvina C. Felix, Karina I. Carvalho, Andreia M. de Matos, José E. Levi, Camila M. Romano, Cristiane C. Centrone, Celia L. de Lima Rodrigues, Expedito Luna, Eric C. M. van Gorp, Albert D. M. E. Osterhaus, Byron E. E. Martina, Esper G. Kallas

**Affiliations:** 1 Viroscience Lab, Erasmus Medical Center, Rotterdam, the Netherlands; 2 Instituto de Medicina Tropical de São Paulo e Departamento de Moléstias Infecciosas e Parasitárias (LIM-52), Faculdade de Medicina, Universidade de São Paulo, São Paulo, Brazil; 3 Department of Infectious Diseases, Hospital Ana Costa, Santos, Brazil; 4 Disciplina de Imunologia Clínica e Alergia (LIM-60), Faculdade de Medicina, Universidade de São Paulo, São Paulo, Brazil; Baylor College of Medicine, United States of America

## Abstract

**Background:**

Severe dengue virus (DENV) disease is associated with extensive immune activation, characterized by a cytokine storm. Previously, elevated lipopolysaccharide (LPS) levels in dengue were found to correlate with clinical disease severity. In the present cross-sectional study we identified markers of microbial translocation and immune activation, which are associated with severe manifestations of DENV infection.

**Methods:**

Serum samples from DENV-infected patients were collected during the outbreak in 2010 in the State of São Paulo, Brazil. Levels of LPS, lipopolysaccharide binding protein (LBP), soluble CD14 (sCD14) and IgM and IgG endotoxin core antibodies were determined by ELISA. Thirty cytokines were quantified using a multiplex luminex system. Patients were classified according to the 2009 WHO classification and the occurrence of plasma leakage/shock and hemorrhage. Moreover, a (non-supervised) cluster analysis based on the expression of the quantified cytokines was applied to identify groups of patients with similar cytokine profiles. Markers of microbial translocation were linked to groups with similar clinical disease severity and clusters with similar cytokine profiles.

**Results:**

Cluster analysis indicated that LPS levels were significantly increased in patients with a profound pro-inflammatory cytokine profile. LBP and sCD14 showed significantly increased levels in patients with severe disease in the clinical classification and in patients with severe inflammation in the cluster analysis. With both the clinical classification and the cluster analysis, levels of IL-6, IL-8, sIL-2R, MCP-1, RANTES, HGF, G-CSF and EGF were associated with severe disease.

**Conclusions:**

The present study provides evidence that both microbial translocation and extensive immune activation occur during severe DENV infection and may play an important role in the pathogenesis.

## Author Summary

The pathogenesis of severe dengue virus (DENV) infection is still not fully understood. It is hypothesized that it is caused by a cytokine storm as is described in severe sepsis. In the sepsis field, the potent immunostimulator lipopolysaccharide (LPS) is proposed to play an important role in the development of a cytokine storm. In a previous study we have found elevated levels of LPS in children with severe DENV infection. In this study we have investigated if we could confirm that microbial translocation occurs in DENV-infected patients. Moreover, we have determined the levels of thirty cytokines to get more insight in the cytokine storm during DENV infections and we have investigated whether microbial translocation is associated with immune activation. The patients in this cohort were classified according to their clinical presentation. Furthermore, a cluster analysis based on the expression of the determined cytokines was applied to identify patients with similar cytokine profiles. With these two techniques, we identified cytokines that may contribute significantly to the cytokine storm, and we could relate elevated levels of LPS to patients with a pro-inflammatory cytokine profile.

## Introduction

Dengue virus (DENV) infection has been emerging in the American and Caribbean region in the past decade. During a DENV-2 outbreak in 2010 in the State of São Paulo, Brazil, more than 34.000 cases and 64 deaths were reported by the Health Department [1]. Symptoms of severe DENV infection range from shock and respiratory distress to major hemorrhagic manifestations and organ failure. The majority of these symptoms are manifest around the time of defervescence. In the early febrile phase, DENV infection is characterized by a high viral load and extensive activation of the Th1 response [2]. Around the time of defervescence, virus titres often decrease below the limit of detection. This critical phase is characterized by extensive immune activation and a so-called cytokine storm (reviewed in [3]) that is characterised by high levels of cytokines with mostly pro-inflammatory properties. The mechanism underlying this cytokine storm is still a matter of debate. Evidence points towards antibody-dependent enhancement in which cross-reactive non-neutralizing antibodies enhance the uptake of virus by monocytic cells (reviewed in [4]). Moreover, it has been proposed that ‘original antigenic sin’ is important in the pathogenesis of dengue (reviewed in [5]), postulating that low-avidity T cells are activated by the virus, but fail to clear it.

During HIV infection, elevated levels of lipopolysaccharide (LPS) were detected in the circulation and correlated with immune activation [6]. There is evidence that a local pro-inflammatory environment in the gut causes disruption of the intestinal barrier, which may eventually result in microbial translocation (MT) (reviewed in [7]). We recently showed that elevated LPS levels are present during DENV infection and correlate with disease severity [8]. DENV replicates in monocytes/macrophages, of which many reside in the gut-associated lymphoid tissue (GALT). Therefore, we hypothesized that this may cause a local pro-inflammatory environment in the bowel, eventually affecting the integrity of the intestinal barrier and resulting in MT. Consequently, because LPS is known to be a potent immune stimulator, elevated LPS levels may contribute to the cytokine storm during severe DENV infection.

In the present study, we studied markers of MT and immune activation in DENV infected patients. Using clinical classification and a cluster analysis we have identified cytokines that correlate with disease severity. Moreover, significantly increased LPS levels were found in a cluster of patients with a pronounced pro-inflammatory cytokine profile.

## Materials and Methods

### Ethics statement

All procedures adopted in this study were performed according to the terms agreed by the Institutional Review Board from the Hospital das Clínicas, University of São Paulo (CAPPesq - Research Projects Ethics Committee). This study was approved by CAPPesq under protocol 0652/09. Written informed consent was obtained from all study volunteers. All included study participants were anonymized with a study number.

### Clinical cohort

This cohort has been described previously [1]. Briefly, during the 2010 outbreak samples were collected from patients with clinical suspected dengue fever presenting at the emergency department, department of internal medicine or the intensive care unit at the Ana Costa Hospital, Santos, State of São Paulo. Patients were diagnosed with DENV infection by detection of DENV NS1 antigen and/or IgM-specific antibodies using a commercially available rapid test (Dengue duo test bioeasy, Standard Diagnostic Inc. 575-34, Korea) or by detection of DENV RNA by real time PCR (RT-PCR). Details concerning the day of onset of fever (day of fever), clinical signs and symptoms and the final diagnosis were recorded by the treating physician. Serum samples were withdrawn and stored at −80°C. Patients were classified according to the 2009 WHO classification [9], [10] and the occurrence of hemorrhagic manifestations and the occurrence of plasma leakage and shock. Hemorrhagic manifestations were observed by the treating physician. The occurrence of plasma leakage was detected by ultrasound or X-ray examination. The diagnosis shock was made by the treating physician based on symptoms such as hypotension, narrow pulse pressure, tachycardia and cold extremities. Age-matched healthy volunteers with a similar socio-economic background were used as controls.

### IgG avidity ELISA and viral load

The IgG avidity test was used to determine primary or secondary DENV infection [11]. Samples with low avidity IgG antibodies were classified as primary DENV infection, whereas samples with high avidity IgG antibodies were classified as secondary. Samples in which IgG antibodies were not detected could not be classified, although the majority was probably primary DENV infection.

Viral load was determined by an “in-house” RT-PCR method and virus serotype was determined by a multiplex PCR. Both methods have been described in detail previously [12]. For both assays RNA was extracted from plasma using the Qiagen Viral RNA kit (Qiagen, Germany). RT-PCRs were conducted in duplicate. For the viral load SuperScript III Platinum SYBR Green One-Step qRT-PCR kit with ROX (Invitrogen, Inc., EUA) and for the dengue serotype multiplex PCR Platinum Taq polymerase (Invitrogen, Brazil) was used. In both RT-PCRs primers covering all four DENV serotypes were used [13]. Sequences of the primers were the following: D1, 5′-TCA ATA TGC TGA AAC GCG CGA GAA ACC G; TS1, 5′- CGT CTC AGT GAT CCG GGG G; TS2, 5′- CGC CAC AAG GGC CAT GAA CAG; TS3, 5′-TAA CAT CAT CAT GAG ACA GAG C; and DENV-4, 5′-TGT TGT CTT AAA CAA GAG AGG TC.

### Markers of MT

Samples were aliquoted and stored at −80°C. Repetitive freeze-thaw cycles were avoided.

LPS was determined with a commercially available Limulus Amebocyte Lysate (LAL) assay (Associates of Cape Cod Incorporated, USA). Samples were diluted 1∶20 with LAL Reagent Water and heat-inactivated at 60°C for 30 minutes. Depyrogenated glassware was used to prevent contamination (Pyrotubes, Associates of Cape Cod Incorporated, USA). Hereafter, 50 µl of LAL was added and samples were incubated in the Pyros Kinetix Flex Machine (Associates of Cape Cod Incorporated, USA). Escherichia coli endotoxin was used to prepare the standard curve. Soluble CD14 (sCD14; ‘Quantikine’ ELISA, R&D Systems, UK), LPS binding protein (LBP ELISA, Hycult Biotech, USA) and IgM and IgG endotoxin core antibodies (EndoCab ELISA, Hycult Biotech, USA) were determined using commercially available assays. The assays were performed according to the manufacturer's instructions and every sample was measured in duplicate. One patient was excluded from the LPS analysis due to extremely high levels of LPS (56504 pg/ml) and therefore a secondary bacteremia could not be excluded.

### Cytokine measurements

Cytokines were measured using a multiplex immunoassay kit with spectrally encoded antibody-conjugated beads (Human Cytokine 30-plex panel, Invitrogen, USA). The following cytokines were measured (Sensitivity limits (pg/ml): EGF (<18,8), Eotaxin (<0), FGF-basic (<12,3), G-CSF (<38,5), GM-CSF (<40), HGF (<0), IFN-α (<116), IFN-γ (<34), IL-1RA (<116), IL-1β (<20), IL-2 (<33), sIL-2R (<40), IL-4 (<108), IL-5 (<40), IL-6 (<13,5), IL-7 (<60), IL-8 (<20), IL-10 (<47), IL-12 (p40/p70) (<40), IL-13 (<60), IL-15 (<58), IL-17 (<80), IP-10 (<640), MCP-1 (<60), MIG (<20), MIP-1α (<17), MIP-1β (<18), RANTES (<20), TNF-α (<21) and VEGF (<823). Serum samples were diluted 1∶2. The test was performed according to the manufacturer's instructions and was run on a Luminex 200 dual laser detection system.

### Cluster analysis

The cluster analysis procedure was adapted from van den Ham et al. [14]. Briefly, cytokine values were log-transformed and subjected to hierarchical correlation clustering (i.e., with distance measure 1 – pearson's pairwise correlation value) using Ward's method that minimizes within-cluster variance. Both patients and cytokines were clustered to obtain a heatmap. Cytokines that had more than 5% of values missing (FGF-basic, GM-CSF, IL-1β, IL-5, IL-7, IL-13 and IL-17) were excluded from the analyses. Three serum samples were excluded from the cytokine analysis, because their levels were out of range for most of the cytokines evaluated and therefore the quality of the sample was most likely compromised. Cluster analysis was performed in R 2.15 (R Development Core Team [R Foundation for Statistical Computing], 2012, www.r-project.org). R scripts used to construct the trees and heatmaps are available upon request.

### Statistical analysis

The Kruskal-Wallis *H* test was used for comparison of more than two groups. Statistical significance between individual groups was determined with the Mann-Whitney *U* test. Using the Bonferroni correction a p-value cut-off of ≤0.0083 for cytokine analyses was applied. For testing the significance of LPS, LBP and sCD14 levels associated with the clinical classifications and the clusters a p-value cut-off ≤0.05 was used. Correlations were calculated using the Spearman's correlation coefficient. To calculate the association of severe disease with the three main clusters the Fisher's exact test was used. For this test we used a p-value cut-off ≤0,05 to reach significance.

## Results

### Cohort

During the 2010 outbreak serum samples were obtained from 811 patients with laboratory confirmed acute DENV infection. From this cohort, 99 patients with non-severe dengue were randomly selected based on the availability of samples and clinical data. Moreover, patients with severe co-morbidity were excluded. Eventually, 50 patients without warning signs (WS−) and 49 with warning signs (WS+) were selected. Only 33 patients presented with severe dengue according to the 2009 WHO case classification [10] and they were all included in this analysis. Among patients with warning signs, 29/49 (59.2%) showed plasma leakage diagnosed by ultrasound/X-rays (pleural and peritoneal 12; peritoneal 12; pleural 5), 23 (46.9%) showed mucosal bleeding, 14 (28.6%) persistent vomiting, 5 (10,2%) abdominal pain and 3 (6,1%) lethargy. Among patients with severe dengue, 27/33 (81,8%) showed signs of severe plasma leakage (25 shock, 2 fluid accumulation leading to respiratory distress), 14 (42,4%) showed severe bleeding and one (3,0%) severe liver involvement (AST and ALT>1000). The clinical presentation and general characteristics of the cohort are described in Table 1.

**Table 1:** Baseline characteristics of the clinical classifications.

2009 WHO dengue case classification			
	WS− (N = 50)	WS+ (N = 49)	Severe (N = 33)
Sex	52% male	61,2% male	39,4% male
Age*	44 (28–57,5)	13,5 (9,25–30,25)	35,5 (15–58,5)
Day of fever*	3 (3–5)	5 (4–7)	6 (4–7)

Plasma leakage and shock			
	No (N = 74)	Plasma leakage (N = 33)	Shock (N = 25)
Sex	52,7% male	66,7% male	32,0% male
Age*	38 (23–55,25)	13 (8–26)	42 (12–62,5)
Day of fever*	4 (3–6)	5 (3–8)	5,5 (4–7)

Hemorrhage			
	No (N = 87)	Minor bleeding (N = 29)	Severe bleeding (N = 16)
Sex	54,0% male	51,7% male	43,8% male
Age*	38 (16–55)	22 (12–59)	31 (9–45)
Day of fever*	4 (3–6)	6 (4–9)	4 (3,5–7)

Baseline characteristics of the cohort when the patients are divided according to the 2009 WHO dengue case classification, the occurrence of plasma leakage and shock and the occurrence of hemorrhagic manifestations. Abbreviations: WS−: non-severe dengue without warning signs, WS+: non-severe dengue with warning signs.

* values are given in median (interquartile range).

Of the 132 patients included, three had a primary and 113 had a secondary infection based on the IgG avidity test [11]. In 16 patients IgG antibodies could not be detected.

### Viral load and dengue serotype

**Figure 1:**
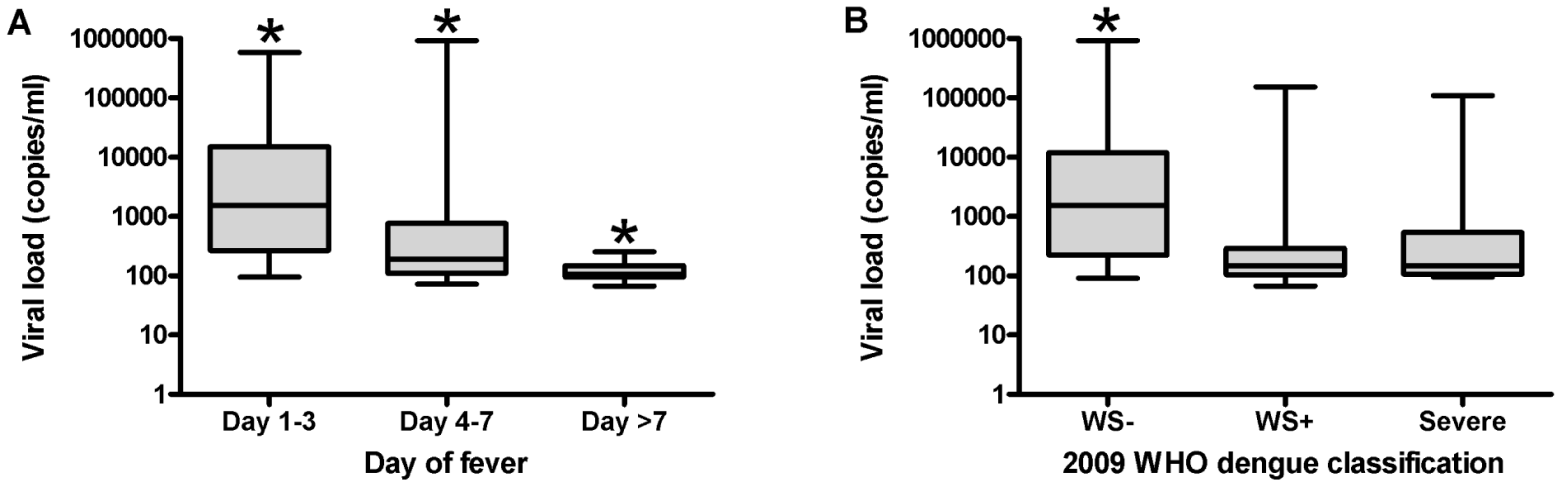
Viral load. A: Day of fever: All three groups differed significantly from each other with the highest levels at day 1–3 and the lowest levels at day>7 (Day 1–3 vs day 4–7 P = 0,001; day 1–3 vs day>7 P<0,0001; day 4–7 vs day>7 P = 0,006). B: 2009 WHO dengue case classification: levels in WS− patients were significantly increased compared to WS+ and severe patients (WS− vs WS+ P<0,0001; WS− vs severe P = 0,001). Abbreviations: WS−: non-severe dengue without warning signs, WS+: non-severe dengue with warning signs. Horizontal bars inside the boxplot indicate the median. The box indicates the interquartile range. Black asterisk = significantly different from all other groups. The Mann-Whitney U test was used to compare the groups with each other.

Viral RNA was detected in 120 of 132 samples. Significantly higher DENV RNA load was detected in samples collected 1–3 days after onset of fever compared to day 4–7 and day >7 (Figure 1). Moreover, DENV RNA levels were significantly higher in WS- patients compared to WS+ and severe patients, but this difference occurred most likely because they presented earlier after the onset of fever (Figure 1, Table 1).

Dengue serotype could be determined in 126/811 (15.5%) patients with laboratory confirmed acute DENV infection during the 2010 Santos outbreak. From these, 118/126 (93.7%) typed as DENV-2, 4 (3.2%) as DENV-1 and 4 (3.2%) as DENV-3. Among the 132 patients included in this study, DENV serotype could be determined in 20 (15.2%) patients. 19 out of 20 patients were typed as DENV-2 and the remaining one as DENV-3.

### Association of levels of circulating cytokines with clinical disease severity

Eotaxin, IL-2, IL-4, IL-1RA and IFN-γ were detected at very low levels in DENV infected patients and healthy controls and did not show any significant differences between groups when patients were classified according to the 2009 WHO classification or the occurrence of plasma leakage/shock and hemorrhage (Table 2). In the majority of samples MIP-1α and TNF-α also showed values below the detection limit. However, some healthy controls showed extremely elevated levels and therefore a significant difference between healthy controls and dengue patients was shown (Table 2).

**Table 2:** Overview of cytokine analyses.

Cytokine	Accession number	2009 WHO classification	Plasma leakage/shock	Hemorrhage	Mortality	Temporal pattern	Cluster analysis	Conclusion
Eotaxin	P51671	--	--	--	--	--	--	No association
MIP-1α (CCL3)	P10147	WS- vs HC P = 0,001 WS+ vs HC P = 0,004	No vs HC P = 0,001 PL vs HC P = 0,006	No vs HC P = 0,001	--	--	↑ cluster A*	Background HC
TNF-α	P01375	WS- vs HC P<0,0001 WS+ vs HC P<0,0001 Sev vs HC P = 0,002	No vs HC P<0,0001 PL vs HC P = 0,002	No vs HC P<0,0001 Minor vs HC P = 0,003	--	--	↑ cluster A*	Background HC
IL-2	P60568	--	--	--	--	--	--	No association
IL-4	P05112	--	--	--	--	--	--	No association
IFN-γ	P01579	--	--	--	--	--	↑ cluster B*	Early antiviral response
IL-1RA	P18510	--	--	--	--	--	↑ cluster C	Association severity in CA
IFN-α	P01562	WS- vs HC P = 0,001 WS+ vs HC P = 0,003 Sev vs HC P = 0,001	No vs HC P = 0,001 PL vs HC P = 0,001 Shock vs HC P = 0,003	No vs HC P = 0,001 Sev vs HC P<0,0001	--	D1-3 vs D4-7 P = 0,002 D1-3 vs D>7 P = 0,003	↑ cluster B*	Early antiviral response
IL-15	P40933	WS- vs HC P<0,0001 WS+ vs HC P<0,0001 Sev vs HC P<0,0001	No vs HC P<0,0001 PL vs HC P<0,0001 Shock vs HC P<0,0001	No vs HC P<0,0001 Minor vs HC P<0,0001 Sev vs HC P<0,0001 No vs Sev P = 0,003	--	--	↓ cluster A ↑ cluster C	Association with dengue in CC and severe disease in CA
IP-10(CXCL10)	P02778	WS- vs HC P<0,0001 WS+ vs HC P<0,0001 Sev vs HC P<0,0001	No vs HC P<0,0001 PL vs HC P<0,0001 Shock vs HC P<0,0001	No vs HC P<0,0001 Minor vs HC P<0,0001 Sev vs HC P<0,0001	--	--	↓ cluster A ↑ cluster C	Association with dengue in CC and severe disease in CA
MIP-1β(CCL4)	P13236	WS- vs HC P = 0,002 WS+ vs HC P<0,0001 Sev vs HC P = 0,002	No vs HC P = 0,001 PL vs HC P = 0,001 Shock vs HC P = 0,004	No vs HC P<0,0001 Minor vs HC P<0,0001	--	D1-3 vs D4-7 P<0,0001 D1-3 vs D>7 P<0,0001	↑ cluster A*	Association dengue in CC. Early antiviral response. Background HC.
MIG(CXCL9)	Q07325	WS- vs HC P<0,0001 Sev vs HC P = 0,002	No vs HC P<0,0001 Shock vs HC P = 0,005	No vs HC P = 0,001 Sev vs HC P<0,0001	--	D1-3 vs D4-7 P = 0,008 D1-3 vs D>7 P = 0,001	↑ cluster B* ↑ cluster C	Early antiviral response. Association severe disease in CA
IL-10	P22301	Sev vs HC P = 0,008	--	--	--	--	↑ cluster C	Association dengue in CC and severe disease in CA.
IL-12	P29459 P29460	WS+ vs HC P = 0,002	--	--	--	D1-3 vs D4-7 P = 0,001 D1-3 vs D>7 P = 0,003	↑ cluster B*	Early antiviral response
G-CSF	P09919	WS- vs Sev P = 0,003	No vs Shock P = 0,002	No vs Sev P<0,0001	P = 0,001	--	↑ cluster C	Association with severe disease in CC and CA
IL-6	P05231	WS- vs Sev P<0,0001 WS+ vs Sev P<0,0001	No vs Shock P<0,0001 PL vs Shock P = 0,001	No vs Sev P = 0,001	P<0,0001	--	↑ cluster C	Highly associated with severe disease
IL-8	P10145	WS+ vs Sev P = 0,004	--	No vs Sev P<0,0001 Minor vs Sev P = 0,002	--	D1-3 vs D4-7 P = 0,008	↑ cluster A* ↑ cluster C	Association with severe disease in CC and CA
RANTES (CCL5)	P13501	WS- vs HC P = 0,001 WS+ vs HC P<0,0001 Sev vs HC P<0,0001 WS- vs Sev P = 0,002	No vs HC P<0,0001 PL vs HC P<0,0001 Shock vs HC P<0,0001 No vs Shock P<0,0001 PL vs Shock P = 0,002	No vs HC P<0,0001 Minor vs HC P<0,0001 Sev vs HC P<0,0001	P = 0,005	--	↓ cluster C	Highly associated with severe disease
EGF	P01133	WS- vs HC P<0,0001 WS+ vs HC P<0,0001 Sev vs HC P<0,0001	No vs HC P<0,0001 PL vs HC P<0,0001 Shock vs HC P<0,0001 No vs Shock P = 0,004	No vs HC P<0,0001 Minor vs HC P<0,0001 Sev vs HC P<0,0001	--	--	↑ cluster A ↓ cluster C	Increased levels background HC. Decreased levels associated with severe disease in CC and CA
HGF	P14210	WS- vs WS+ P = 0,005 WS- vs Sev P = 0,001	No vs Shock P = 0,001	--	--		↑ cluster C	Associated with severe disease in CC and CA
MCP-1(CCL2)	P13500	WS+ vs HC P = 0,008 WS- vs WS+ P = 0,001	--	--	--	D1-3 vs D4-7 P<0,0001 D1-3 vs D>7 P<0,0001	↑ cluster B* ↑ cluster C	Early antiviral response. Association severe disease in CC and CA
VEGF	P15692	--	--	--	--	--	↑ cluster C	Association with severe disease in CA
IL-2R	A2N4P8	--	--	Sev vs HC P = 0,003 No vs Sev P = 0,002 Minor vs Sev P = 0,008	P = 0,005	--	↑ cluster C	Highly associated with severe disease, hemorrhage in particular.

Overview of the significant associations of all analyses performed and specified per cytokine. The Mann-Whitney U test was used to compare the groups with each other. Abbreviations: HC: healthy control. 2009 WHO classification: WS-: non-severe dengue without warning signs, WS+: non-severe dengue with warning signs, Sev: severe dengue. Plasma leakage/shock: No : no plasma leakage occured, PL: plasma leakage detected, Hemorrhage: No : no hemorrhagic symptoms, Minor: minor hemorrhage, Sev: severe hemorrhage. Temporal pattern: D1-3: day 1-3 after the onset of fever, D4-7: day 4-7 after the onset of fever, D>7: more than 7 days after the onset of fever. Cluster analysis: * only in a part of the cluster this cytokine is up- or downregulated. Conclusions: CC: clinical classification, CA: cluster analysis.

IFN-α, IL-10, IL-12 IL-15, IP-10, MIG and MIP-1β were significantly increased or decreased in dengue patients compared to healthy controls if patients were classified according to the 2009 WHO classification (Table 2, Figure S1). These cytokines did not show significant differences among the disease severity groups.

**Figure 2:**
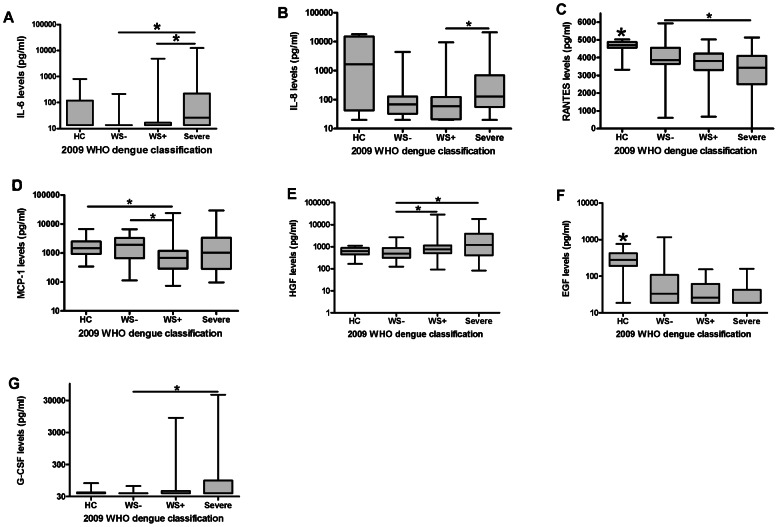
Cytokine levels in dengue virus infected patients classified according to the 2009 WHO dengue classification. A: IL-6: Levels in severe patients are significantly increased compared WS− (P<0,0001) and WS+ (P<0,0001) patients (KW P<0,0001, KW dengue groups P<0,0001). B: IL-8: Levels in severe patients are significantly elevated compared to WS+ patients (P = 0,004) (KW P = 0,004, KW dengue groups P = 0,011). C: RANTES: Levels in severe (P = 0,002) patients are significantly decreased compared to WS− patients. Levels in all patient groups are significantly decreased compared to HC (WS− vs HC P = 0,001, WS+ and severe vs HC P<0,0001), (KW P<0,0001, KW dengue groups P = 0,006). D: MCP-1: Levels in WS+ patients are significantly decreased compared to WS− (P = 0,001) patients and HC (P = 0,008) (KW P = 0,006, KW dengue groups P = 0,006). E: HGF: Levels in severe (P = 0,001) and WS+ (P = 0,005) patients are significantly increased compared to WS-patients (KW P = 0,001, KW dengue groups P = 0,001). F: EGF: Levels in all patient groups are significantly decreased compared to HC (WS−, WS+ and severe vs HC P<0,0001, KW P<0,0001, KW dengue groups P = 0,03). G: G-CSF: Levels in severe patients are significantly increased compared to WS- patients (P = 0,003, KW P = 0,02, KW dengue groups P = 0,008). Legend: HC = healthy control, WS− = non-severe dengue without warning signs. WS+ = non-severe dengue with warning signs, KW = kruskal wallis. Horizontal bars inside the boxplot indicate the median. The box indicates the interquartile range. Black asterisk = significantly different from all other groups. Underlined black asterisk = significant difference between two groups. The Mann-Whitney U test was used to compare the groups with each other.

Some cytokines showed significant differences in levels between dengue disease severity groups. Using the 2009 WHO dengue case classification, levels of RANTES and MCP-1 were significantly increased in WS− patients compared to patients with severe and WS+ dengue respectively. In contrast, levels of IL-6, IL-8, HGF and G-CSF were significantly increased in severe dengue compared to uncomplicated disease (Table 2, Figure 2).

**Figure 3:**
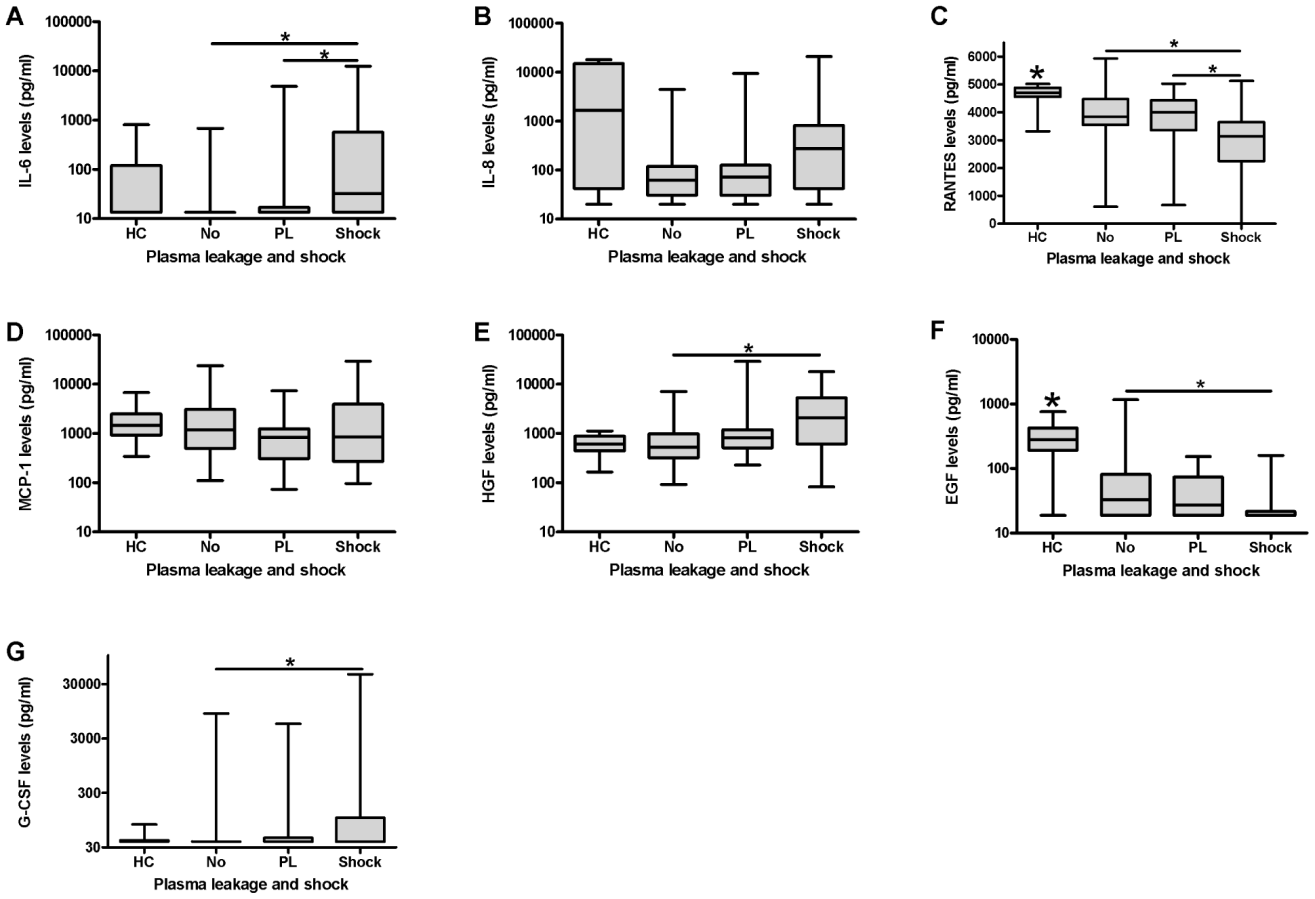
Cytokine levels in dengue virus infected patients classified according to the occurrence of plasma leakage/shock. A: IL-6: Levels in shock patients are significantly increased compared to patients with (P = 0,001) and without (P<0,0001) plasma leakage (KW P<0,0001, KW dengue groups P<0,0001). B: IL-8: No significant differences (KW P = 0,01, KW dengue groups P = 0,04). C: RANTES: Levels in shock patients are significantly decreased compared to patients with (P = 0,002) and without (P<0,0001) plasma leakage. Levels in all patient groups are significantly decreased compared to HC (No, PL and shock vs HC P<0,0001, KW P<0,0001, KW dengue groups P = 0,001). D: MCP-1: No significant differences (KW P = 0,162, KW dengue groups P = 0,17). E: HGF: Levels in patients with shock are significantly increased compared to patients with no plasma leakage (P = 0,001) (KW P = 0,001, KW dengue groups P = 0,001). F: EGF: Levels in shock patients are significantly decreased compared to patients without (P = 0,004) plasma leakage. Levels in all patient groups are significantly decreased compared to HC (No, PL and shock vs HC P<0,0001, KW P<0,0001, KW dengue groups P = 0,015). G: G-CSF: Levels in patients with shock are significantly elevated compared to patients without plasma leakage (P = 0,002, KW P = 0,01, KW dengue groups P = 0,004). Abbreviations: HC =  healthy control, No =  no occurrence of plasma leakage, PL = plasma leakage, KW = kruskal wallis. Horizontal bars inside the boxplot indicate the median. The box indicates the interquartile range. Black asterisk = significantly different from all other groups. Underlined black asterisk = significant difference between two groups. The Mann-Whitney U test was used to compare the groups with each other.

When these cytokines were determined in patients classified according to the occurrence of plasma leakage and shock, levels of RANTES and EGF were significantly decreased in patients with shock compared to patients with uncomplicated dengue. Moreover, levels of IL-6, HGF and G-CSF were significantly increased in shock patients compared to patients with uncomplicated disease (Table 2, Figure 3).

Patients were also classified according to the occurrence of hemorrhage. Levels of sIL-2R, IL-6, IL-8, IL-15 and G-CSF were significantly increased in patients with severe bleeding compared to patients with no bleeding (Table 2, Figure S2).

**Figure 4:**
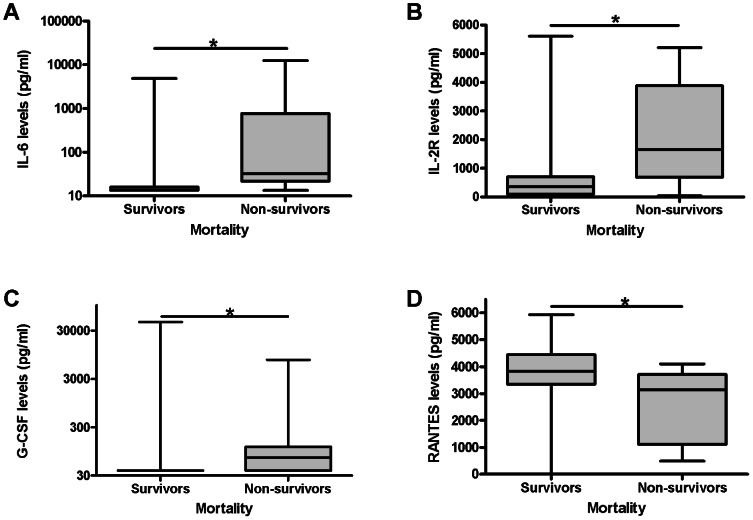
IL-6, IL-8, IL-2R and RANTES are associated with mortality. A: IL-6: Levels are significantly elevated in non-survivors compared to survivors (P<,0,0001). B: IL-2R: Levels are significantly elevated in non-survivors compared to survivors (P = 0,005). C: G-CSF: Levels are significantly elevated in non-survivors compared to survivors (P = 0,001). D: RANTES: Levels are significantly decreased in non-survivors compared to survivors (P = 0,005). Underlined black asterisk = significant difference between two groups. The Mann-Whitney U test was used to compare the groups with each other.

Nine out of 132 patients died within 14 days after the onset of fever. In these patients IL-6, G-CSF and sIL-2R were significantly increased and RANTES significantly decreased in non-survivors compared to survivors (Table 2, Figure 4).

IFN-α, IL-12, MCP-1, MIG, MIP-1β showed a dynamic temporal pattern during the course of disease. They were significantly increased at day 1–3 after the onset of fever compared to day 4–7 and day>7 (Table 2, Figure S3). This may explain why the levels of MCP-1 were significantly higher in uncomplicated than in more severe dengue in patients classified according to the 2009 WHO dengue case classification, since patients with non-severe dengue presented earlier in their course of disease (Table 1). Interestingly, the mediators IFN-α (P = 0.001), IL-12 (P = 0.01), MCP-1 P<0.0001), MIG (P = 0,01) and MIP-1β (P<0.0001) showed to have a significant positive correlation with the viral load (data not shown).

### Cluster analysis identifies a group of patients with a pro-inflammatory cytokine profile

**Figure 5:**
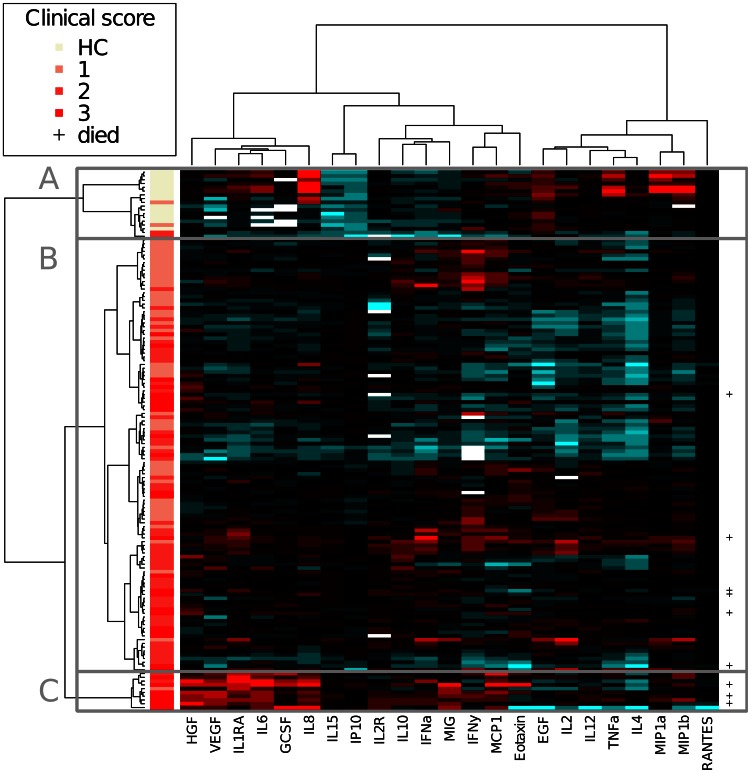
Heatmap of cluster analysis. A cluster analysis was performed with 23 cytokines, which resulted in a dendrogram indicated on the left of the heatmap. Every horizontal line indicates one patients. The vertical bar on the left of the heatmap indicates the disease severity of the patient. A: Cluster with mainly healthy controls and four dengue patients. B: Cluster with mild to moderately ill dengue virus infected patients. C: Cluster with severely ill dengue virus infected patients. Abbreviations: 1: non-severe dengue without warning signs. 2: non-severe dengue with warning signs. 3: severe dengue. +: patient died within 14 days after the onset of fever.

The cluster analysis groups samples or cytokines based on cytokine levels only, and not based on clinical presentation (non-supervised analysis). The sample and cytokine cluster analyses can be combined and visualized as a heatmap (Figure 5). A dendrogram shows the similarity between samples (left side of figure 5), where samples in the same branch are more similar regarding their cytokine profiles to each other than to samples in other branches. The sample dendrogram can be divided into three principle clusters that largely segregate healthy controls (cluster A), mild to moderately ill DENV infected patients (cluster B), and severely ill DENV infected patients (cluster C). Clinical disease was more severe in cluster C than in clusters A and B, illustrated by a statistically significant higher incidence of severe disease (P = 2,2×10^−16^), shock (3,4×10^−5^), severe hemorrhage (P = 0,007) and death (P = 0,03) in this cluster compared to cluster A and B (Table 3).

**Table 3:** Clinical characteristics of the cluster analysis.

Cluster	A				B				C				Fisher's exact test
	N = 18				N = 115				N = 10				
Age (years)*	26,5 (22–35)				31,5 (13–49)				45 (14–63)				
Sex	38,9% male				53,0% male				30,0% male				
Day of fever*	6 (4–11)				4 (3–6)				4 (3–5)				
2009 WHO dengue case classification	77,8,0% (N = 14) HC	11,1% (N = 2) WS−	11,1% (N = 2) WS+			40,0% (N = 46) WS−	39,1% (N = 45) WS+	20,9% (N = 24) Severe		10,0% (N = 1) WS−	20,0% (N = 2) WS+	70,0% (N = 7) Severe	Severe dengue P = 2,2×10^−16^
Survival	77,8,0% (N = 14) HC	22,2% (N = 4) Survived				94,8% (N = 109) Survived	5,2% (N = 6) Died			70,0% (N = 7) Survived	30,0% (N = 3) Died		Shock P = 3,4×10^−5^
Hemorrhage	77,8,0% (N = 14) HC	16,7% (N = 3) NO	5,6% (N = 1) Minor			68,7% (N = 79) NO	22,6% (N = 26) Minor	8,7% (N = 10) Severe		40,0% (N = 4) NO	20,0% (N = 2) Minor	40,0% (N = 4) Severe	Severe hemorrhage P = 0,007
Plasma leakage and shock	77,8,0% (N = 14) HC	16,7% (N = 3) NO	5,6% (N = 1) PL			57,4% (N = 66) NO	27,0% (N = 31) PL	15,7% (N = 18) Shock		10,0% (N = 1) NO	20,0% (N = 2) PL	70,0% (N = 7) Shock	Death P = 0,03

Clinical manifestations of patients divided in the three clusters. Abbreviations: HC: healthy control, WS−: non-severe dengue without warning signs, WS+: non-severe dengue with warning signs, PL: plasma leakage.

* values are given in median (interquartile range).

A subgroup of ‘healthy control’ cluster A displayed elevated levels of inflammatory cytokines when compared to other control samples, including MIP-1α, MIP-1β, TNF-α, EGF and IL-8. These controls are likely to have suffered from an underlying unidentified inflammatory condition (Table 2, Figure 5).

The majority of dengue cases were part of cluster B. In cluster B 40% of patients suffered from WS−, 39% from WS+ and 21% from severe dengue (Table 3). This distribution resembles the whole cohort, which consisted of 38% WS−, 37% WS+ and 25% severe dengue. The cytokine pattern in this cluster shows a rather diffuse pattern. A few patients show increased concomitant expression of IFN-γ, IFN-α, MCP-1, MIG and IL-12, which is indicative for an early antiviral response.

Cluster C shows a strong pro-inflammatory cytokine pattern. RANTES and EGF are downregulated, whereas IL-6, IL-8, IL-10, IL-15, IL-1RA, sIL-2R, HGF, VEGF, G-CSF, MCP-1, IP-10, and MIG are upregulated compared to other clusters. Interestingly, IL-1RA, IL-10, IL-15, IP-10, MIG and VEGF are associated with severe disease in the cluster analysis, but not with severe disease using the clinical classifications. Using for example the 2009 WHO classification IL-10, IL-15, IP-10 and MIG were significantly elevated in dengue patients compared to healthy controls, but levels were not significantly elevated in severe compared to uncomplicated dengue. Cluster C identifies a group of patients with an extensive pro-inflammatory cytokine profile, suggestive for a cytokine storm. Moreover, severe clinical symptoms occurred significantly more often in cluster C compared to the other clusters.

### MT is associated with severe dengue

**Figure 6:**
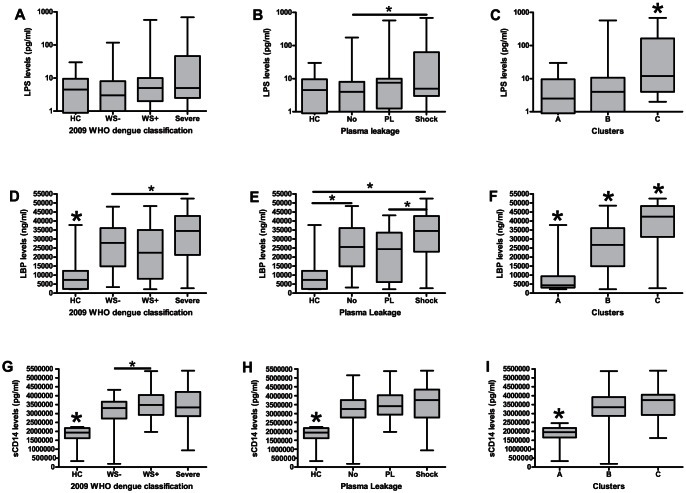
LPS, LBP and sCD14 levels in dengue virus infected patients. LPS: A: No significant differences in the 2009 WHO dengue case classification. B: Levels in patients with shock were significantly increased compared to patients without plasma leakage (P = 0,04). C: Cluster analysis: cluster C was significantly elevated compared to cluster A (P = 0,01), and B (P = 0,02). LBP: D: Levels were significantly elevated in all dengue patients compared to HC (WS− vs HC P = 0,009, WS+ vs HC P = 0,03, Severe vs HC P = 0,01). Levels in patients with severe dengue were significantly elevated compared to WS− dengue (P = 0,03). E: Levels were elevated in patients with shock (P = 0,008) and no plasma leakage (P = 0,008) compared to HC. Levels were also elevated in patients with shock compared to patients with plasma leakage (P = 0,03). F: In the cluster analysis levels in cluster C were significantly elevated compared to cluster A (P = 0,002) and B (P = 0,007). Moreover, cluster B was significantly elevated compared to cluster A (P<0,0001). sCD14: G: In the 2009 classification levels of sCD14 in DENV infected patients were significantly elevated compared to HC (WS−, WS+ and severe vs HC P<0,0001). Levels were significantly increased in WS+ compared to WS− patients (P = 0,04). H: In the plasma leakage/shock classification levels of sCD14 in DENV infected patients were significantly elevated compared to HC (No, PL and shock vs HC P<0,0001). I: In the cluster analysis cluster B (P<0,0001) and C (P = 0,002) were significantly elevated compared to cluster A. Abbreviations: HC : healthy control, WS−: non-severe dengue without warning signs. WS+: non-severe dengue with warning signs, No : No occurrence of plasma leakage, PL: occurrence of plasma leakage. Horizontal bars inside the boxplot indicate the median. The box indicates the interquartile range. Black asterisk = significantly different from all other groups. Underlined black asterisk = significant difference between two groups. The Mann-Whitney U test was used to compare the groups with each other.

Statistically significant elevated LPS levels were found in cluster C compared to ‘dengue’ cluster B and ‘healthy control’ cluster A (Figure 6). In the 2009 classification there proved to be a trend towards higher LPS levels in severe dengue, although these differences were not statistically significant. However, in the plasma leakage/shock classification LPS levels were significantly increased in patients with shock compared to patients with no plasma leakage. In the 2009 classification, LBP levels were significantly increased in dengue patients compared to healthy controls. Moreover, levels in severe patients were significantly increased compared to WS− patients. In the plasma leakage/shock classification levels were significantly increased in patients with shock and no plasma leakage compared to healthy controls. Moreover, levels in patients with shock were significantly increased compared to patients with plasma leakage. In the cluster analysis LBP levels in all three clusters differed significantly from each other. sCD14 levels were significantly increased in DENV infected patients compared to healthy controls in the 2009 and the plasma leakage/shock classification and in the ‘dengue’ clusters B and C compared to the ‘healthy control’ cluster A. Moreover, in the 2009 classification levels in WS+ patients were significantly increased compared to WS− patients. When patients were classified according to the occurrence of hemorrhagic manifestations, LPS levels were not significantly different, and sCD14 and LBP again showed to be significantly elevated in DENV infected patients compared to controls (Data not shown). No significant differences in IgM- and IgG-specific endotoxin core antibodies were found among the groups classified according to the 2009 classification or the occurrence of plasma leakage and shock (Data not shown).

## Discussion

In this study, we have examined a cohort of dengue patients and healthy controls to investigate the role of immune activation and MT in DENV pathogenesis. We found evidence for the occurrence of MT during DENV infection. Furthermore, in the cluster analysis, we showed that the cluster of patients with the highest LPS levels appeared to suffer from a cytokine storm.

The two complementary analysis techniques applied in this study yielded similar results. However, the cluster analysis identified more markers associated with severe disease than the clinical classification system. The cluster analysis groups patients based on the occurrence of identical inflammatory processes, overcoming the potential clinical classification biases that may occur due to the fact that disease presentation of patients can be quite variable and the severity of disease is subject to clinical interpretation. In the cluster analysis, levels of cytokines determined the outcome of the clusters. Therefore this technique cannot be used to relate absolute values of cytokines to the clusters with patients. Altogether, the strength of our approach is the use of both clinical classification and cluster analysis in order to increase the sensitivity to find markers of disease severity.

One limitation of this study is the cross-sectional study design. We have recorded the disease severity of the patient at the time of inclusion and at the same moment the samples for LPS and cytokine analysis were drawn, so the levels of LPS and cytokines were related to signs and symptoms that were present at that same time.

Both in a previous [8] and in the present study, we have shown that elevated levels of LPS are associated with severe dengue. Moreover, MT was indirectly confirmed by increased levels of LBP and sCD14 as observed in sepsis patients [15], [16]. In contrast to our previous study, the association between LPS levels and clinical disease severity was less strong. However, also in this study there proved to be a significant association in patients classified according to the occurrence of plasma leakage and shock. Moreover, LPS levels were significantly increased in the cluster with the highest incidence of shock (cluster C) and levels of LBP did also show a direct association with disease severity in the 2009 and the plasma leakage/shock classification. This cohort differed from our previous study in several ways: age of the population (children vs. children and adults), the geographical location (Indonesia vs. Brazil) and the samples used (plasma vs. serum). Whether age or different pathogen pressures at different geographical locations may account for the observed differences remain to be established. IgM and IgG endotoxin core antibodies were determined, but no strong association with disease severity was found. This is in agreement with studies in HIV and sepsis patients, which show conflicting results with regards to the association between endotoxin core antibodies and disease severity [6], [17], [18].

It has been hypothesized that severe DENV infection is caused by an exaggerated immune response, associated with a cytokine storm (reviewed in [3], [19]). The exact definition of a cytokine storm is still a matter of debate. In general it is assumed that a cytokine storm starts with an excessive release of pro-inflammatory cytokines (e.g. TNF-α and IL-1β). These cytokines then induce other pro-inflammatory (e.g. IL-6), but also anti-inflammatory cytokines (e.g. IL-10). This augmented immune response could therefore be the result of a disturbed balance between pro- and anti-inflammatory cytokines. During severe DENV infections a cytokine storm has been proposed to be responsible for the increased vascular permeability and coagulation disturbances (reviewed in [19]).

Studies in patients with HIV and visceral leishmaniasis showed that MT may contribute to severe disease through excessive immune activation [6], [20]. It is known that LPS stimulation can induce the production of IL-6, IL-8, TNF-α and IL-1β [21] and the growth factors VEGF and HGF [22]. Interestingly, in the present study high levels of four of these markers were found in the pro-inflammatory cluster C. This suggests that MT may play a role in the cytokine storm in severe dengue. Moreover, Bosisio et al. [23] showed that priming of mononuclear cells with IFN-γ increased the expression of the TLR4 receptor and subsequent LPS-induced cytokine production. This would suggest that DENV induced IFN-γ production could enhance the pro-inflammatory LPS signaling pathway. In addition, Chen et al. [24], [25] showed that LPS could prolong DENV infection of monocytes and macrophages. A sustained DENV infection due to MT may also contribute to the cytokine storm during DENV infection. All these studies suggest that MT may play an important role in the initiation and perpetuation of the cytokine storm during severe DENV infection. However, in this study MT was associated with extensive immune activation, but to investigate whether there is a causal relationship between MT and the cytokine storm further studies are warranted.

Our cohort confirms several known associations for dengue. In agreement with previous work, our study showed evidence of a strong Th1 response in the early phase of disease with peak levels of IFN-α [26], [27], IL-12 [28], [29], [30], MCP-1 [31], [32], [33], MIG and MIP-1β [31]. All these Th1 cytokines correlated significantly with viral load, suggesting that they are associated with a host response aiming at reducing the viral load.

In the present study we have quantified pro- and anti-inflammatory mediators to provide evidence for a role of a cytokine storm in severe dengue patients. Levels of IL-10, IL-15, VEGF, G-CSF and IP-10 were increased in ‘severe dengue’ cluster C in the cluster analysis. High levels of IL-10 and VEGF have been described in severe dengue, especially at the day of defervescence [2], [28], [29], [34], [35]. Interestingly, patients in severe cluster C presented around this time (day 3–5 after onset of fever). The high incidence of shock in cluster C could be partly explained by high levels of VEGF and MCP-1, which are proposed to be important contributors to plasma leakage [33], [35]. IL-15 and IP-10 [36] were reported to play an important role in the NK cell response, whereas G-CSF [37] stimulates neutrophil development and differentiation. High levels of IL-15, IP-10 and G-CSF in cluster C suggest that extensive activation of the innate immune system may contribute to the cytokine storm in severe dengue. In contrast, high levels of IL-10 have an inhibitory effect on dendritic cells and macrophages (reviewed in [38]).

In both the clinical classifications and the cluster analysis, IL-6, IL-8, sIL-2R, RANTES, HGF and EGF were strongly associated with severe disease. High levels of IL-6 and IL-8 were reported in dengue cases with severe plasma leakage and shock [39] and in non-survivors [40], [41], [42]. IL-6 production is induced by TNF-α and IL-1β [37]. In our study no increased levels of TNF-α and IL-1β were found. This is in agreement with earlier reports [31], [34], [39], [42], [43] and can be explained by the observation that TNF-α and IL-1β are produced early after infection and are removed quickly from the circulation. In addition, sIL-2R has been associated with severe dengue [2], [27], [43] and is proposed to serve as a marker of immune activation (reviewed in [44]). Thrombocytopenia is a hallmark of DENV infection and since thrombocytes are an important source of RANTES and EGF, severe thrombocytopenia may explain the depletion of these two markers. This has been described previously in severe dengue [28] and cerebral malaria [45].

In summary, we provide evidence that MT is associated with extensive immune activation during severe dengue. LPS may play an important role in the development of the cytokine storm. Besides the classical mediators (e.g. IL-6, IL-8, IL-10), we identified cytokines (IL-1RA, sIL-2R), chemokines (MCP-1, IP-10, MIG, RANTES) and growth factors (HGF, EGF, G-CSF, VEGF) that may play an important role in the cytokine storm during severe DENV infection.

## Supporting Information

Checklist S1 **STROBE Checklist.** STROBE checklist for this study.
(DOC)

Figure S1 **Cytokine levels in dengue virus infected patients are significantly different compared to healthy controls.** **A: IFN-α:** Levels in DENV infected patients are significantly elevated compared to HC (WS− vs HC P = 0,001, WS+ vs HC P = 0,003 and severe vs HC P = 0,001, KW P = 0,005, KW dengue groups P = 0,93). **B: IL-15:** Levels in DENV infected patients are significantly elevated compared to HC (WS−, WS+ and severe vs HC P<0,0001, KW P<0,0001, KW dengue groups P = 0,08). **C: IP-10:** Levels in DENV infected patients are significantly elevated compared to HC (WS−, WS+ and severe vs HC P<0,0001, KW P<0,0001, KW dengue groups P = 0,82). **D: MIP-1β:** Levels in dengue patients are significantly decreased compared to HC (WS− vs HC P = 0,002, WS+ vs HC P<0,0001, Sev vs HC P = 0,002, KW P = 0,002, KW dengue groups P = 0,31). **E: MIG:** Levels in WS− (P<0,0001) and severe (P = 0,002) patients are significantly elevated compared to HC (KW P<0,0001, KW dengue groups P = 0,03). **F: IL-10:** Levels in severe patients are significantly elevated compared to HC (P = 0,008, KW P = 0,08, KW dengue groups P = 0,54). **G: IL-12:** Levels in WS+ patients are significantly decreased compared to HC (P = 0,002, KW P = 0,05, KW dengue groups P = 0,26). Abbreviations: HC = healthy control, WS− = non-severe dengue without warning signs. WS+ = non-severe dengue with warning signs. Horizontal bars inside the boxplot indicate the median. The box indicates the interquartile range. Black asterisk = significantly different from all other groups. Underlined black asterisk = significant difference between two groups. The Mann-Whitney U test was used to compare the groups with each other.
(EPS)

Figure S2 **Cytokine levels in dengue virus infected patients classified according to the occurrence of hemorrhage.** **A: IL-6:** Levels are significantly elevated in patients with severe bleeding compared to patients with no hemorrhage (P = 0,001) (KW P = 0,007, KW dengue groups P = 0,003). **B: IL-8:** Levels in patients with severe bleeding are significantly elevated compared to patients with minor (P = 0,002) and no (P<0,0001) hemorrhage (KW P = 0,001, KW dengue groups P = 0,001). **C: IL-15:** Levels in patients with severe hemorrhage are significantly elevated compared to patients with no hemorrhage (P = 0,003). Levels in HC are significantly decreased compared to all other dengue groups (WS−, WS+ and severe vs HC P<0,0001) (KW P<0,0001, KW dengue groups P = 0,009). **D: sIL-2R:** Levels are significantly elevated in patients with severe bleeding compared to HC (P = 0,003) or patients with minor (P = 0,008) or no hemorrhage (P = 0,002) (KW P = 0,013, KW dengue groups P = 0,007). **E: G-CSF:** Levels in patients with severe hemorrhage are significantly increased compared to patients with no hemorrhage (P<0,0001) (KW P = 0,005, KW dengue groups P = 0,002). Abbreviations: HC  =  healthy control, No = No occurrence of hemorrhage. KW = kruskal wallis. Horizontal bars inside the boxplot indicate the median. The box indicates the interquartile range. Black asterisk = significantly different from all other groups. Underlined black asterisk = significant difference between two groups. The Mann-Whitney U test was used to compare the groups with each other.
(EPS)

Figure S3 **Levels of cytokines during the course of disease.** **A: IFN-α:** Levels at day 1–3 were significantly increased compared to day 4–7 (P = 0,002) and day>7 (P = 0,003) (KW P = 0,001). **B: MCP-1:** Levels at day 1–3 were significantly increased compared to day 4–7 (P<0,0001) and day>7 (P<0,0001) (KW P<0,0001). **C: MIG:** Levels at day 1–3 were significantly increased compared to day 4–7 (P = 0,008) and day>7 (P = 0,001) (KW P = 0,001). **D: MIP-1β:** Levels at day 1–3 were significantly increased compared to day 4–7 (P<0,0001) and day >7 (P<0,0001) (KW P<0,0001). **E: IL-12:** Levels at day 1–3 were significantly increased compared to day 4–7 (P = 0,001) and day >7 (P = 0,003) (KW P = 0,001). Abbreviations: KW = kruskal wallis. Black asterisk = significantly different from all other groups. The Mann-Whitney U test was used to compare the groups with each other.
(EPS)
